# Daily Nutritional Intake of Pediatric Patients (N = 64) on Extracorporeal Membrane Oxygenation from 2018 to 2022: A Single-Center Report

**DOI:** 10.3390/nu15143221

**Published:** 2023-07-20

**Authors:** Megan Brackmann, Annika Lintvedt, Benjamin Kogelschatz, Erika Heinze, Jessica L. Parker, Karen Ferguson, Elizabeth Rosner, Brian Boville, Mara L. Leimanis-Laurens

**Affiliations:** 1 Department of Pediatrics and Human Development, College of Human Medicine, Michigan State University, Grand Rapids, MI 49503, USA; brackma8@msu.edu (M.B.); lintvedt@msu.edu (A.L.); benkogelschatz@gmail.com (B.K.); elizabeth.rosner@helendevoschildrens.org (E.R.); brian.boville@helendevoschildrens.org (B.B.); 2 Pediatric Critical Care Medicine, Helen DeVos Children’s Hospital, Grand Rapids, MI 49503, USA; karen.ferguson@corewellhealth.org; 3 Corewell Health, Grand Rapids, MI 49503, USA; erikaheinze58@gmail.com (E.H.); jessica.parker2@corewellhealth.org (J.L.P.)

**Keywords:** extracorporeal membrane oxygenation, nutritional needs, length of stay, mortality, caloric and protein goals, pediatric intensive care unit

## Abstract

Nutrition in pediatric populations who require life-saving extracorporeal membrane oxygenation (ECMO) remains a debate. We sought to identify if nutritional needs were met in a patient cohort. A retrospective chart review of patients (N = 64) requiring ECMO at Helen DeVos Children’s Hospital between 2018 and 2022 was evaluated for demographics, daily nutritional data, laboratory values, ECMO complications, and outcome data, with primary outcome measures of percent protein and percent caloric intake. Secondary outcome measures included the intensive care unit length of stay, time on ECMO, mortality, and day 1 severity of illness scores (Pediatric Logistic Organ Dysfunction). The timeline partially overlapped with the COVID-19 pandemic. Data were collected for 467 ECMO days with a median age of 2.6 months; 57.8% of patients were male and 65.6% were with one pre-existing comorbidity. Venoarterial (VA) ECMO was utilized in 84.4% of patients; the ECMO indication was cardiac in 53.1% of patients. The 28-day mortality was 43.8%. The proportion of days in which the caloric goal was met was 0%; the proportion of days in which protein goals were met was 33.3%. Non-cardiac ECMO patients had a greater number of days where caloric goals were met (*p*-value = 0.04). Mortality at 28 days was not statistically significant (*p*-value = 0.28) for calories or protein administered. The patient cohort struggled to meet calorie and protein goals while on ECMO.

## 1. Introduction

Extracorporeal membrane oxygenation (ECMO) is a potentially life-saving cardiopulmonary support system reserved for critically ill patients who experience failed conventional therapies. Blood from the venous system is circulated outside of the body where it passes through an oxygenation device, which functions to saturate hemoglobin with oxygen and extract carbon dioxide. The blood is then re-warmed and returned to either the venous or arterial circulation. Typically, when blood is returned to the venous system (venovenous, i.e., VV-ECMO), the patient receives respiratory support but remains dependent on their own hemodynamic control. On the other hand, blood returned to the arterial circulation (venoarterial, i.e., VA-ECMO) bypasses both the heart and lungs and provides hemodynamic stability in addition to respiratory support [1]. ECMO can be further stratified into the following sub-groups: 1. neonatal; 2. pediatric; 3. adult; and 4. ECPR (ECMO used in CPR for patients with cardiac arrest [2]). The Extracorporeal Life Support Organization (ELSO) Registry Report from 2022 demonstrated that the use of ECMO continues to steadily increase. Neonatal survival was between 70 and 90% whereas pediatric survival ranged from about 60 to 70% [3]. A retrospective cohort study (2005–2016) conducted at a single tertiary care institution on 160 patients (105 neonatal intensive care unit (NICU) patients, 55 pediatric intensive care unit (PICU) patients) observed that the median time on ECMO was 13 days plus/minus 11 days. Sixty-six percent of the patients experienced a complication related to ECMO with neurologic problems including a hemorrhage and seizures being the most common [4]. Other complications in ECMO patients include bleeding, infection, mechanical issues, thrombosis, and nutritional deficiencies [1]. 

Estimating the energy expenditure and protein needs of pediatric patients on ECMO is challenging. Critically ill patients are typically in a stress response, hypercatabolic state, which increases their energy expenditure and protein requirements [5], while requiring sedatives and paralytics, which decreases their caloric needs [6]. Standard equations used to calculate a child’s resting energy expenditure (REE) fail to accurately predict the value in the majority of critically ill children [7], and performing indirect calorimetry, considered the gold standard for determining caloric expenditure in this population, is contraindicated for patients on ECMO. A study of 203 patients showed that the underfeeding of either energy or protein occurred in a third of patients [8]. Adequate protein delivery has been proven to decrease mortality and increase ventilator-free days [9]. One study went so far as to suggest that for every gram of daily protein ingested, there was a suggested 1% reduction in mortality [10]. Overfeeding calories has been associated with complications such as steatosis and liver dysfunction [6]. 

Research has shown that the effective delivery of nutrition can positively impact outcomes including mortality [11]. Few studies have provided reliable data to further nutritional goals while on ECMO [12]. This study seeks to identify whether nutritional needs were met in a patient cohort regarding their daily caloric and protein goal. The secondary objective was to observe how the timing and content of feeding are related to several outcomes including mortality, the ICU length of stay, and the time on ECMO. 

## 2. Materials and Methods

### 2.1. Nutritional Assessment

#### 2.1.1. Rounding and Response to Consults

Multidisciplinary rounds with an Attending MD, Fellow MD, Resident MD, Nurse Practitioner (NP), bedside registered nurse (RN), pharmacist, and Registered Dietitian (RD) occurred daily for three pediatric intensive care teams; however, on weekends, there was no pharmacist or RD available for rounding. Two RDs shared responsibility for all three rounds, which occurred simultaneously and were conducted outside each patient’s room with the family member(s) or guardian(s) invited to participate. 

A comprehensive nutrition assessment, including a nutrition focused physical exam, when appropriate, was completed in response to all consults that originated from several sources—medical staff (Advanced Practice Providers, nursing) and criteria met by the RD screening per our Medical Nutrition Therapy Standards of Care. Nursing consults are a result of an indicator being present upon admission for the nutrition risk screening criteria (Supplemental Information S1–S4). On Saturdays, one RD screened newly admitted patients and responded to new consult orders entered by 12:00 at a minimum, often completing those that came in by 14:00. The same RD was on call on Sunday but performed assessments based on only a few diagnoses (eating disorder, failure to thrive, malnutrition, inborn errors of metabolism/metabolic disorder) unless contacted by the medical/surgical team with a request to do so. All nutrition assessments were typically completed within 24 h but no later than 48 h of the order being placed or initiation of nutrition support therapy.

#### 2.1.2. Initiation and Advancement of Nutrition Support

The initiation, advancement, and type of nutrition support chosen are guided by The Provision and Assessment of Nutrition Support Therapy in the Pediatric Critically Ill Patient: Society of Critical Care Medicine (SCCM) and American Society for Parenteral and Enteral Nutrition (ASPEN) [7]. Every effort is made to initiate enteral nutrition within 24–48 h of admission. In most cases, enteral feedings are advanced based on an established guideline in the PICU (Supplemental Information S3), but exceptions do occur. The initial rate of infusion of enteral feeds may start at a lower rate, or feeds may start at the rate per the guideline but be advanced more slowly than every 4 hours based on the patient’s medical/surgical condition. When the goal for the nutrition prescription using a pediatric or adult formula is for a higher concentration (1.5 calories per mL) than a standard concentration (1.0 or 1.06 calories per mL), feedings are typically advanced in one of two ways. Either the rate of delivery is advanced based upon the PICU Enteral Feeding Guideline to the desired goal rate followed by increasing the formula to a higher concentration as the final step, or feeds are started at a higher concentration and the rate of delivery is advanced more slowly than the guideline to take into account the increased concentration. When advancing breastmilk or standard infant formula with a goal of a higher concentration than 20 calories per ounce, feedings are advanced using one of the same two approaches. There is no formal guideline for the advancement of enteral feeds for cardiac PICU patients, but these feedings are typically advanced as follows. For normal advancement, if the weight is less than 3 kg, feeds begin at 1 mL per hour and advance by 1 mL per hour every 6 h. If the weight is greater than 3 kg, feeds begin at 2 mL per hour and advance by 2 mL per hour every 6 h. For high-risk advancement, if the weight is less than 3 kg, feeds begin at 1 mL per hour and advance by 1 mL per hour every 12 h. If the weight is greater than 3 kg, feeds begin at 1 mL per hour and advance by 1 mL per hour every 6 h.

#### 2.1.3. Determination of Nutrition Goals

ASPEN provides goals for caloric and protein provision for critically ill children and initiation and advancement guidelines when using parenteral nutrition. For determining calorie and protein needs, we followed ASPEN guidelines for determining energy needs for patients aged 3 to 18 years old (Schofield × 1.0). For patients aged 0 to up to 3 years, the ASPEN guideline for a goal of Schofield × 1.0 provides approximately the equivalent caloric delivery as 60% of the dietary reference intake (DRI). When changing to this guideline (shortly after the guidelines were published in 2017), we informally observed a trend of weight loss when changing from using a goal of 90% of the DRI to using this method for calculating caloric needs. This prompted a change in practice back to using a goal of 90% of the DRI for caloric needs. A weight loss trend was not observed in patients aged 3 to 18 years of age with the change in practice from using Schofield × 1.2 to using Schofield × 1.0.

Our goal was for patients to receive 90% of their estimated needs for calories and protein with enteral feeds within 24 h and each day after but this was more difficult to achieve for pediatric cardiac intensive care unit (PCICU) patients than PICU patients, given the fluid restriction often in place for post-operative cardiac patients (Supplemental Information S4). While the goals for calories and protein are the same whether a patient is cardiac or not, the guidelines for advancing feeds differ as post-op cardiac patients are generally at a higher risk for intolerance, hence the slower advancement of feeds. Cardiac patients are also fluid restricted to a much greater extent, which also contributes to delays in reaching feeding goals. For the purposes of the study, we chose the target energy intake of 67% based on SCCM/ASPEN recommended guidelines. Patients were divided into 3 groups based on the percentage of goal calories and protein met: 0–33% (Group 1), 34–66% (Group 2), and 67% or greater. No patients fell into the category of 67% or greater.

### 2.2. Study Design

#### 2.2.1. Site, Patient Population

All pediatric and neonatal patients who required ECMO at Helen DeVos Children’s Hospital (HDVCH) from 2018 to 2022 were included in this retrospective chart review (N = 64). The patient’s charts were accessed, and data were collected from Epic from 30 beds (~1800 admissions per year unit). Subjects were divided into three groups: (1) Pediatric Cardiac ECMO, (2) Pediatric non-Cardiac ECMO, and (3) Neonatal ECMO. VV and VA ECMO were separated. 

#### 2.2.2. Data Extraction, Management, and Quality Control

A multitude of variables were collected: general patient descriptors including name, age, gender, race/ethnicity, weight at hospital admission (kg), body mass index (BMI), medical record number (MRN), PICU admission, diagnosis and weight (kg), ECMO start date and time, type of ECMO (VV or VA), and indication for ECMO (cardiac vs. non-cardiac). They qualified as binary, ordinal, integer, or numeric. Nutrition data included type of nutrition (enteral, parenteral, both, or NPO), content of the feed if enteral, caloric prescription (kcal/d), caloric intake, percent caloric intake of the goal, protein prescription (gm/d), protein intake, percent protein intake of the goal, whether nutritional goals were met, whether they were overfed or underfed and weight (kg), and were extracted from a 24 h time period (7:00 a.m. to 6:59 a.m. the following day). Multiple laboratory values were automatically extracted from the chart and included sodium, potassium, bicarbonate, blood urea nitrogen (BUN), creatinine, glucose, total and ionized calcium, total bilirubin, and albumin (these are not reported in the final draft of the manuscript). Specific details of the ECMO course were further collated along with ECMO type, pump, clinical indication, cardiac arrest in the 24 h prior to ECMO initiation, whether the patients have been on ECMO prior to during the hospitalization, and ECMO complications.

Data were managed utilizing REDCap^®^ software—a secure, web-based, data management system designed for clinical research. Information regarding study subjects was kept confidential and managed according to the Health Insurance Portability and Accountability Act of 1996 (HIPAA). 

There were four data collectors for 32 patients in round one and two data collectors for an additional 32 patients in round two. Variables collected manually include ECMO pump type, ECMO indication, cardiac arrest in the 24 h prior to ECMO initiation, prior ECMO history, patient comorbidities, daily weight, feed type, route, timing, calories, protein, REE, extra protein, bowel movement, nutritional goals met, over/underfed, vasopressors, weight at end of PICU stay, ECMO complications, mortality at 28 days, discharge location, and route of feeds upon discharge. Additional variables were extracted automatically from Virtual Pediatric Systems (VPS). 

Following round one of data collection, a comprehensive audit was performed to assess the accuracy of the data extraction. The cut-off for repeating the data collection process was 90%. Each data collector was randomly assigned multiple patients (~5% (3 patients) of the total) and were tasked to review each manual data extraction variable and provide a score of one (entered correctly) or zero (entered incorrectly) within an Excel spreadsheet and subsequently change the data entry in REDCap^®^ if necessary. The final audit showed 95% accuracy and therefore data collection was not repeated.

### 2.3. Data Analysis

#### Outcome Measures

Outcome measures included PICU length of stay; hospital length of stay; total duration of parenteral nutrition (PN); total duration of enteral nutrition (EN); time to PN; time to EN; days on mechanical ventilation; length of ECMO run; ECMO free days; ECMO complications, including infection (proven by culture), cannula site bleeding, a pulmonary hemorrhage, renal failure (evidenced by creatinine), an arrhythmia, central nervous system (CNS) infarction, a seizure, or death; comorbidities; weight at end of PICU stay (kg); 28-day mortality; severity of illness (PELOD) score; and secondary organ dysfunction.

Numeric data were expressed as the mean ± standard deviation or median [25th, 75th percentile], whichever was most appropriate for the data point. Categorical data were expressed as the frequency (percent). When looking at two group comparisons on outcomes that were numeric, an independent *t*-test was used or a Wilcoxon Rank Sum, depending on assumptions. When looking at more than two group comparisons (e.g., pediatric cardiac and pediatric non-cardiac vs. neonate) with numeric outcomes, a One-Way ANOVA or Kruskal–Wallis test were utilized, dependent on assumptions being met. If the outcome was categorical, a Chi-square Test or Fisher’s Exact Test was utilized. A multiple linear regression model was used to model the percent of the calorie goal met with several other independent variables. Backwards selection with a *p*-value cut-off of 0.1 was used to create the final model. All other analyses were assessed at the 0.05 level. 

## 3. Results

### 3.1. Patient Demographics, ECMO Specifics, Hospital Information

Nutrition data were collected over 467 total days from a consecutive series of ECMO patients, of which 19 were neonatal and 45 were pediatric patients with a median age of 2.6 months. The cohort was 57.8% male and 67.2% White, with 65.6% of patients having at least one pre-existing comorbidity (the highest reported being congenital heart disease in n = 33 (51.6%)). Median patient BMI is reported as 13.5, with median admission severity of illness scores (PELOD) at 13. Patient demographics are summarized in Table 1. 

VA ECMO was utilized in 84.4% of patients (15.6%, VV ECMO). The ECMO indication was cardiac in 53.1% of patients. Cardiac arrest in the 24 h prior to ECMO initiation occurred in 45.3% of patients. Half of the patients had an ECMO complication and these were categorized as mechanical (n = 9; 14.1%); hemorrhagic (n = 6; 9.5%); neurologic (n = 19; 30%); renal (n = 10; 15.7%); cardiovascular (n = 10; 15.6%); pulmonary (n = 3; 4.8%); and limb (n = 3; 4.8%) (Table 2). While we acknowledge that continuous renal replacement therapy (CRRT) can spuriously lower creatinine, this occurred in only 9.4% of our patients in the study. It may have caused an underestimation of peak creatinine in these patients. 

From ECMO characteristics for our patient population, we reviewed the hospital course for the patient cohort (Table 3). The median hospital LOS was 31 days [16, 81], and the median PICU LOS was 20 days [12, 52]. The median ECMO run length was 5 days [3, 9]. ECMO-free days were defined as 28 days minus the number of ECMO days, which was 23 [20, 25] days. Necrotizing enterocolitis (NEC) was reported in 5 (7.9%) of patients. The 28-day mortality of the patient cohort was 28 (43.8%). Patients were discharged home (n = 17; 26.6%), to the hospital floor (n = 10; 15.6%), or to a long-term facility (n = 8; 12.5%). Discharge details for patients include those with feeding tubes (n = 19; 30.2%); a ventilator (n = 3; 4.8%); tracheostomy (n = 2; 3.2%), and long-term TPN (n = 2; 3.2%). The weight at the end of the PICU stay was 7.4 [4.1, 19.5] kg, with a wide range and variabilities (fluid retention, use of diuretics) that may be clinically relevant.

### 3.2. Summary of Nutrition Information

#### 3.2.1. Nutrition for ECMO Days

**Figure 1:**
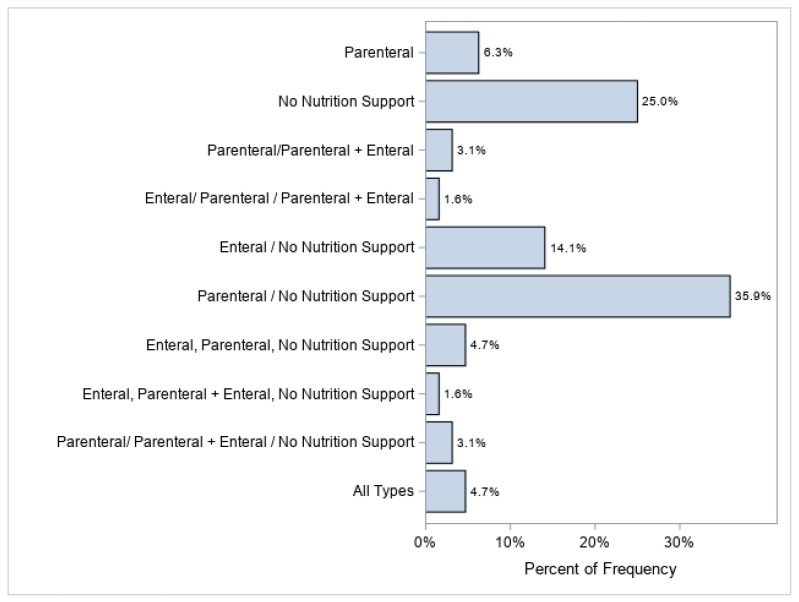
Feeding type over all ECMO runs. This is the distribution of the combinations each patient may have had during their stay, i.e., 4 (6.3%) patients only received parenteral nutrition during their stay, whereas 3 (4.7%) patients had a combination of all feed types during their stay. Data are expressed as the count (percent). The type(s) of feeding patients received included parenteral nutrition via a central line (with or without lipids), enteral nutrition delivered to the gastrointestinal tract (most often using a nasogastric tube), and/or neither parenteral nor enteral nutrition (no nutrition support). “All Types” represents patients that received parenteral nutrition, enteral nutrition, parenteral + enteral, and no nutrition support.

Daily nutritional information was extracted from the electronic medical record (EMR) and summated over the entire ECMO course. This information was initially recorded as feeding types, as presented in Figure 1. The most frequent combination included patients with some amount of parenteral nutrition/no nutritional support (n = 23; 35.9%), followed by no nutrition support (n = 16; 25%), and enteral/no nutritional support (n = 9; 14.1%). The breakdown of calories vs. protein relative to goals met was further broken down and summarized (Supplemental Table S1).

**Figure 2:**
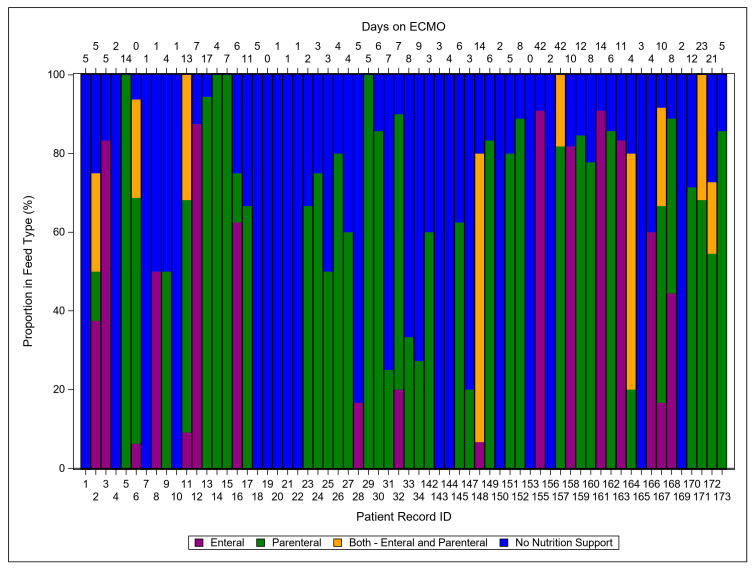
Proportion of feed type for days of ECMO (N = 64). Note: patients with “0” were on ECMO for less than 24 h.

Total feeds over all days on ECMO are summarized in Figure 2, which illustrates all nutritional feeds over time, color coded for enteral (purple), parenteral (green), enteral and parenteral (yellow), and no nutritional support (blue). This heatmap was created with the intention to visualize totals for patients 1–34 and 142–173, as well as for the total of 64 patients. 

#### 3.2.2. Nutrition by ECMO Variables of Interest: Cardiac, Mortality

Upon further review, we deemed it necessary to divide the study subjects into groups, in part given that the cardiac patients and non-cardiac patients adhered to different feeding guidelines (as described earlier) as an independent variable (Table 4). Cardiac–ECMO patients vs. non-cardiac ECMO patients were not statistically different when comparing feed type, hospital LOS, PICU LOS, and days on mechanical ventilation (DMV). The proportion of days in which the caloric goal was met in cardiac–ECMO patients was 0% vs. non-cardiac ECMO patients, where the proportion of days in which the protein goal was met was 33.3%. Therefore, non-cardiac ECMO patients had a greater number of days where their caloric goals were met (*p*-value = 0.04), which was found to be statistically significant. Mortality at 28 days was not statistically significant (*p*-value = 0.28) for total calories or protein administered in our single-center investigation.

Additionally, groups were analyzed according to mortality as an outcome (dependent variable) (Supplemental Table S2). There were no statistically significant findings when analyzing for differences in calories or grams of protein delivered, the proportion of days on extra protein, the proportion of days where calorie and protein goals were met, the overall percent of the calorie goal (including patients with 0 calories), or the percent of the calorie goal met for patients that received greater than 0 of the goal. Zero patients met either the calorie or protein goal of receiving at least 67% of calculated needs, resulting in 100% that were underfed. 

#### 3.2.3. Nutritional Information by Groups Regarding 0–33% and 34–66% of Goals Met during ECMO Course

Patients were divided into three groups based on the percentage of goal calories and protein met: 0–33% (Group 1), 34–66% (Group 2), and 67% or greater. No patients fell into the category of 67% or greater. Group 1 vs. Group 2 for the median ECMO run length resulted in a significant difference (*p* < 0.0001) between the two calorie groups. More specifically, the median ECMO run length for Group 1 was found to be lower than Group 2 (2 vs. 7) (Table 5 and Table 6).

Group 1 vs. Group 2 for ECMO free days (defined as 28 days minus days on ECMO) suggested a significant difference (*p* < 0.0001) between the two calorie groups. More specifically, the median number of ECMO free days in Group 1 was higher than Group 2 (26 vs. 22).

There was a significant association between patient age and the percentage of the calorie goals met. For each month increase in patient age, the percent of the calorie goal met increased by 0.1% while holding TPN dependence constant. There was also a significant association between TPN dependence and the percentage of the caloric goals met. Patients with TPN dependence had 28.25% more of their percent calorie goal met than patients without TPN dependence, when holding age constant (Table 7). We looked at the R-square (0.1309), AIC (375.0), and Mallows C(p) (~1.5). The AIC and Mallows C(P) were lowest for the final model after removing all the other variables for backwards regression. While this model is not ideally fit, the assumptions were met and may not have been achieved due to the smaller N (n used in model is 60), and therefore findings cannot be generalized until more research is completed.

## 4. Discussion

The work presented summarizes observational data from a pediatric patient cohort from 2018 to 2022. Together, we describe both cohort characteristics as well as outcome measures for this representative consecutive series of patients. Zero patients met either the calorie or protein goal of receiving at least 67% of calculated needs, resulting in 100% who were underfed. Reaching nutritional goals for this patient population is challenging, and although the initial goal of this work was to determine time to enteral feeds, instead we were able to review different goal points, which revealed surprising results. A proportion of the patients did not obtain feeds during their ECMO course. Upon further review, patients who did not obtain any feeds beyond 48 h included patients with shock bowel (bowel segments with marked submucosal edema and intense mucosal enhancement) [13], feeding intolerance, prior bowel surgery, failure to thrive, and cardiac shock (secondary to COVID-19), to name a few. 

While not describing an ECMO population specifically, the concept of withholding TPN feeds has been reported in the sentinel multicenter randomized control trial of Fivez et al., which described a superior outcome for critically ill children (for feeds withheld longer than 1 week; n = 717), as compared to early feeds (less than 1 week; n = 723) [14]. Furthermore, although enteral feeds were provided along with micronutrients, infection rates were lower in the patient population with late TPN (10.7% vs. 18.5%). Therefore, determining “minimal essential nutritional intake” in the pediatric ECMO population requires further evaluation.

Patients with a cardiac indication for ECMO received fewer and had a lower amount of calories than non-cardiac ECMO patients. Nutritional assessments for cardiac–ECMO patients follow a different feeding guideline as described earlier. More recent work documents that early EN is not associated with harm for patients with cardiogenic or obstructive shock (N = 220), who require at least 2 days of VA-ECMO [15]. 

We did not find differences in mortality. Secondly, ECMO as an “exposure” was not found to be correlated to nutritional intake. What takes place at the bedside is a complex conversation for the clinical team of which the dietitian is a key player. Providing adequate nutrition remains a significant challenge in caring for pediatric patients on ECMO. One of the main issues lies in the interruption of feeds as reported in 90% of patients receiving VV-ECMO in adult populations, which is further segmented to patients requiring procedures at the bedside (40%) and with GI intolerances (23%) (which includes vomiting, concerns over abdominal distention and constipation, high gastric residual volume, high stool output) [8]. 

This study was partially conducted in the timeframe of the coronavirus disease 2019 (COVID-19) pandemic. Therefore, it is both appropriate and necessary to address the complexities of nutrition research in this context. A meta-analysis performed in 2022 analyzed survival rates of patients with COVID-19 on ECMO. This large-scale study deemed ECMO as a potentially beneficial intervention in patients with COVID-19 with an overall mortality rate of 39%. This, however, was reported as higher than the overall mortality rate in patients with influenza utilizing ECMO (relative risk, 1.34; *p* = 0.03) [16]. 

One interesting finding was that the patients who were on ECMO longer had greater total caloric and protein intake. From previous reports looking at untargeted lipidomics in our single-center investigations, we had found that ECMO patients had a greater nutritional intake than patients with multi-organ dysfunction syndrome alone (not requiring ECMO), as stratified over three time points during their PICU admission (baseline, 48 h, prior to PICU discharge or 8 days) [17]. We attributed this to a greater hemodynamic stability in ECMO patients.

Optimizing the delivery of calories and protein is another topic that requires refinement. As Figure 2 illustrates, there is heterogeneity in feeds that exist for this patient population, as well as several patients for whom, over the PICU ECMO course, no nutrition was delivered. Traditionally, parenteral routes have been utilized for pediatric and neonatal ECMO patients. However, there is growing literature to support that enteral feeds may be more beneficial [18]. It has been shown that an early (<2 days) initiation of enteral feeding resulted in a significantly lower in-hospital mortality as well as a lower 28-day mortality than a similar group with a late (>2 days) initiation of enteral nutrition [15,19]. These same benefits were not seen with patients started on parenteral feeds upon the initiation of ECMO; however, there does appear to be a benefit to supplemental late parenteral nutrition. A group of pediatric patients demonstrated shorter PICU stays and fewer infections when late parenteral nutrition (day 8) was used as a supplement to enteral feeds versus a group where parenteral feeds were initiated within 24 h of admission [7]. Generally, the new guidelines suggest that enteral nutrition is preferred as a starting point, and if the patient’s nutritional goals are not being met, then parenteral delivery can be used as a supplement. 

Specifically, patients with congenital heart disease (CHD) are a large population who require ECMO support. In these patients, when hemodynamically stable, EN is still considered a safe nutritional route [20]. Despite these recommendations, nutritional adequacy remains low in children on ECMO. Five-hundred patients were analyzed across 31 PICUs and researchers found a widespread inadequacy of both energy (34% prescribed) and protein (35% prescribed) via EN. Common barriers to proper delivery included the interruptions of feeds secondary to procedures, intolerance, and fluid restriction due to a volume overload [21]. Though EN is preferred, there are contraindications for EN including hemodynamic instability (defined as an increasing lactate and/or vasoactive requirement), an unrepaired congenital diaphragmatic hernia (CDH), significant ileus, or other severe abdominal pathology [11]. In these cases, PN should be utilized to decrease energy and protein deficits [11].

Limitations include the lack of reporting for feed delays or interruptions. However, anecdotally, especially for enterally fed patients, there would have been at least one interruption. Provider bias also exists among Intensivists as well as regarding when to hold feeds and involves a very involved and detailed chart audit as there is no particular specified entry for feeding interruptions in the EMR. Patients receiving <24 h of ECMO could have been removed from the analysis. Additional analytical attempts to determine outcome stratification could have involved looking at patients with 0–48 h, 49–96 h, and over 96 h on ECMO. However, the group sizes would certainly have become sparse in number. One resolution is to attempt a multi-site collaboration, whereby we might be able to increase the sample size and increase our statistical power.

## 5. Conclusions

Our patient cohort struggled to meet calorie and protein goals while on ECMO, suggesting current feeding guidelines need to be modified for this population. Determining the appropriate nutrition goals while on ECMO and its relationship to outcomes will help clinicians personalize nutrition plans for these critically ill patients.

## Figures and Tables

**Table 1:** Patient Demographics (N = 64).

Variable	Overall (N = 64)
Neonatal vs. Pediatric	
Neonatal	19 (29.7)
Pediatric	45 (70.3)
Age (months)	2.6 [0.0, 59.8]
Gender (Male)	37 (57.8)
Race/Ethnicity	
White or Caucasian	43 (67.2)
Black or African American	9 (14.1)
Hispanic	1 (1.6)
Asian	1 (1.6)
Other	5 (7.8)
Not Reported/Unknown	5 (7.8)
Ethnicity	
Non-Hispanic	55 (85.9)
Hispanic	4 (6.2)
Unknown	5 (7.8)
BMI	N = 58; 13.5 [11.3, 16.8]
Day 1 PELOD Score	N = 49; 13 [11, 23]
Does the patient have comorbidities?	42 (65.6)
Prematurity	10 (15.6)
Congenital Heart Disease	33 (51.6)
Chromosomal Abnormality	11 (17.2)
Feeding Intolerance	14 (21.9)
G- or J-Tube Dependence	9 (14.1)
TPN Dependence	3 (4.7)
Short Gut	0 (0.0)
Liver Failure	0 (0.0)
Failure to Thrive	7 (10.9)
Prior Bowel Surgery	1 (1.6)

Notes: comorbidities do not add up to total patients because some patients could have had more than one; therefore, the proportion per comorbidity and the percentages could add up to more than 100%. BMI: basal metabolic index; PELOD: Pediatric Logistic Organ Dysfunction; TPN: total parenteral nutrition. Numeric data are expressed as the median [25th, 75th percentile] and categorical data are expressed as the count (percent).

**Table 2:** ECMO Information.

Variable	Overall (N = 64)
ECMO Type	
VA	54 (84.4)
VV	10 (15.6)
ECMO Pump	
Centrifugal	20 (31.2)
Roller	44 (68.8)
ECMO Indication	
Cardiac	34 (53.1)
Non-Cardiac	30 (46.9)
Cardiac Arrest in the 24 h prior to ECMO Initiation	29 (45.3)
Has the patient been on ECMO prior to during this hospitalization?	2 (3.1)
Were there any ECMO Complications?	32 (50.0)
Mechanical: Clots: Hemofilter	1 (1.6)
Mechanical: Circuit Change	1 (1.6)
Mechanical: Thrombosis/Clots: Circuit Component	7 (10.9)
Hemorrhagic: GI Hemorrhage	1 (1.6)
Hemorrhagic: Surgical Site Bleeding	1 (1.6)
Hemorrhagic: Peripheral cannulation site bleeding	1 (1.6)
Hemorrhagic: Mediastinal cannulation site bleeding	3 (4.7)
Neurologic: Brain Death	2 (3.1)
Neurologic: Seizures: Clinically determined	2 (3.1)
Neurologic: Seizures confirmed with EEG	4 (6.7)
Neurologic: CNS Infarction (US, CT, or MRI)	2 (3.1)
Neurologic: Intraventricular CNS hemorrhage (US, CT, or MRI)	2 (3.1)
Neurologic: Intra/Extra-parenchymal CNS hemorrhage (US, CT, or MRI)	5 (7.8)
Neurologic: CNS Diffuse Ischemia (CT/MRI)	2 (3.1)
Renal: Creatinine, 1.5–3.0	3 (4.7)
Renal: Creatinine >3.0	1 (1.6)
Renal: Renal Replacement Therapy Required	6 (9.4)
Cardiovascular: CPR Required	6 (9.4)
Cardiovascular: Cardiac Arrhythmia	4 (6.2)
Pulmonary: Pneumothorax requiring treatment	2 (3.1)
Pulmonary: Pulmonary Hemorrhage	1 (1.6)
Limb: Ischemia	1 (1.6)
Limb: Fasciotomy	1 (1.6)
Limb: Amputation	1 (1.6)

Note: CNS: central nervous system; CPR: cardiopulmonary resuscitation; CT: computerized tomography; ECMO: extracorporeal membrane oxygenation; EEG: electroencephalogram; GI: gastrointestinal; MRI: magnetic resonance imaging; US: ultrasound; VA: venoarterial; VV: venovenous. Data are expressed as the count (percent).

**Table 3:** Hospital Information.

Variable	Overall (N = 64)
Hospital LOS (days)	31 [16, 81]
PICU LOS (days)	20 [12, 52]
ECMO Run Length (days)	5 [3, 9]
ECMO Free Days	23 [20, 25]
Weight at End of PICU Stay (kg)	7.4 [4.1, 19.5]
Any Evidence of NEC	N = 63; 5 (7.9)
Mortality (28 Days)	28 (43.8)
Discharge Location from PICU	
Home	17 (26.6)
Hospital Floor	10 (15.6)
Long-Term Facility	8 (12.5)
Other	29 (45.3)
Were they discharged with feeding tubes?	N = 63; 19 (30.2)
Were they discharged on a ventilator?	N = 63; 3 (4.8)
Were they discharged with a trach?	N = 63; 2 (3.2)
Were they discharged on long-term TPN?	N = 63; 2 (3.2)

Notes: ECMO: extracorporeal membrane oxygenation; LOS: length of stay; NEC: necrotizing enterocolitis; PICU: pediatric intensive care unit; TPN: total parenteral nutrition; trach: tracheostomy. Numeric data are expressed as the median [25th, 75th percentile] and categorical data are expressed as the count (percent).

**Table 4:** Nutrition Information/Outcomes by ECMO Indication: Cardiac vs. Non-Cardiac.

Variable	Cardiac (N = 34)	Non-Cardiac (N = 30)	*p*-Value
Feed Type Same Across ECMO Days	8 (23.5)	10 (33.3)	0.3840
Parenteral	1 (12.5)	3 (30.0)	
No Nutrition Support	7 (87.5)	7 (70.0)	
No Calories Present	9 (26.5)	7 (23.33)	0.7724
Calories/Proteins Present	25 (73.5)	23 (76.7)	0.7724
Total Calories	947.70 [307.00, 2220.30]	3391.30 [1112.30, 4290.90]	0.0431
Total Protein	55.04 [14.80, 146.10]	121.40 [30.74, 250.00]	0.1732
Proportion of Days on Extra Protein	57.32 [20.00, 75.00]	40.45 [22.22, 71.43]	0.3826
Proportion of Days Calorie Goal Met	0.00 [0.00, 0.00]	0.00 [0.00, 11.11]	0.0399
Proportion of Days Protein Goal Met	N = 34 25.00 [0.00, 57.14]	N = 29 33.33 [0.00, 54.55]	0.8872
Overall Percent of Calorie Goal (including patients with 0)	N = 32 21.2 [3.25, 42.3]	N = 28 43.11 [7.8, 53.3]	0.1368
Percent of Calorie Goal (patients that received >0 of goal)	N = 25 32.76 [16.11, 46.98]	N = 23 47.87 [27.84, 51.31]	0.0906
Mortality (28 Days)	17 (50)	11 (36.7)	0.2833
Hospital Length of Stay	36 [16, 96]	22.5 [15, 54]	0.2337
PICU Length of Stay	27.1 [14.1, 51.1]	17.4 [8.8, 54.6]	0.4634
Days on Mechanical Ventilation	15.7 [6.7, 67.38]	14.7 [9.8, 34.7]	1.0

Note: ECMO: extracorporeal membrane oxygenation; PICU: pediatric intensive care unit. Numeric data are expressed as the median [25th, 75th percentile] and categorical data are expressed as the count (percent).

**Table 5:** Nutrition Information (Calorie Groups).

Variable	Overall (N = 60)	Group 1 (N = 12)	Group 2 (N = 48)	*p*-Value
Number of Days in ICU	22 [13, 54]	18 [6, 36]	25 [14, 56]	0.2715
Numbers of days on Vent	N = 30; 15 [9, 48]	N = 7; 9 [2, 68]	N = 23; 16 [10, 48]	0.22
ECMO run length	5 [3, 10]	2 [2, 4]	7 [4, 11]	<0.0001
ECMO free days	23 [19, 25]	26 [25, 27]	22 [17, 24]	<0.0001
Hospital Length of Stay	33 [18, 87]	26 [8, 45]	37 [19, 94]	0.1742
Mortality (Yes)	24 (40)	5 (41.7)	19 (39.6)	1.0 *
Cardiac (Yes)	32 (53.3)	7 (58.3)	25 (52.1)	0.698

Calorie Groups: Group 1: 0–33% of calorie goal met; Group 2: 34–66% of calorie goal met; and Group 3: ≥67% of calorie goal met (note: no patients fell into this category). Numeric data are expressed as the median [25th, 75th percentile] and categorical data are expressed as the count (percent). * Fisher’s Exact Test was used.

**Table 6:** Nutrition Information (Protein Groups).

Variable	Overall (N = 58)	Group 1 (N = 10)	Group 2 (N = 48)	*p*-Value
Number of Days in ICU	22 [14, 53]	18 [10, 34]	25 [14, 56]	0.23
Numbers of days on Vent	N = 30; 15 [9, 48]	N = 7; 9 [2, 68]	N = 23; 16 [10, 48]	0.22
ECMO run length	6 [3, 10]	2 [2, 3]	7 [4, 11]	<0.0001
ECMO free days	23 [18, 25]	26 [25, 26]	22 [17, 24]	<0.0001
Hospital Length of Stay	33 [18, 82]	26 [12, 35]	37 [19, 94]	0.1355
Mortality (Yes)	23 (39.7)	4 (40)	19 (39.6)	1.0 *
Cardiac (Yes)	32 (55.2)	7 (58.3)	25 (52.1)	0.4865

Protein Groups: Group 1: 0–33% of protein goal met; Group 2: 34–66% of protein goal met; and Group 3: ≥67% of protein goal met (note: no patients fell into this category). Numeric data are expressed as the median [25th, 75th percentile] and categorical data are expressed as the count (percent). * Fisher’s Exact Test was used.

**Table 7:** Multiple Linear Regression Model—Percent Feed Goal Met.

Label	Parameter Estimate	95% Confidence Limits		*p*-Value
Intercept	24.33	17.32	31.34	<0.0001
Patient Age	0.10	0.013	0.19	0.0250
Comorbidities (choice = TPN dependence)	28.26	1.53	54.98	0.0386

Note: TPN: total parenteral nutrition.

## Data Availability

Data are available upon request.

## Supplementary Materials

The following supporting information can be downloaded at: https://www.mdpi.com/article/10.3390/nu15143221/s1, Supplemental Information S1: Screening for Nutrition Risk, Determination of Nutritional Goals; Table S1: Nutrition Information; Table S2: Nutrition Information by Mortality; Table S3: Multiple Linear Regression Model—Percent Feed Goal Met; Table S4: Regression models with Outcomes—Percent Feed Goal Met (Independent Variable). Supplemental Information S2: Enteral Feeding Guideline; Supplemental Information S3: Enteral Feeds’ Addendum; Supplemental Information S4: CHOP Feeding protocol.

## Abbreviations

ASPEN: American Society of Parenteral and Enteral Nutrition; BMI: body mass index; CDH: congenital diaphragmatic hernia; CHD: congenital heart disease; CNS: central nervous system; CPR: cardiopulmonary resuscitation; CRRT: continuous renal replacement therapy; CT: computerized tomography; DMV: days on mechanical ventilation; DRI: dietary reference intake; ECMO: extracorporeal membrane oxygenation; EEG: electroencephalogram; ELSO: Extracorporeal Life Support Organization; EMR: electronic medical record; EN: enteral nutrition; GI: gastrointestinal; HDVCH: Helen DeVos Children’s Hospital; LOS: length of stay; MRI: magnetic resonance imaging; MRN: medical record number; NEC: necrotizing enterocolitis; NP: nurse practitioner; PCICU: pediatric cardiac intensive care unit; PELOD: Pediatric Logistic Organ Dysfunction; PICU: pediatric intensive care unit; PN: parenteral nutrition; RD: registered dietician; REE: resting energy expenditure; RN: registered nurse; SCCM: Society of Critical Care Medicine; TPN: total parenteral nutrition; US: ultrasound; VA: venoarterial; VAD: ventricular assist device; VPS: virtual pediatric systems; VV: venovenous.
