# Supplementary material for: Daily Nutritional Intake of Pediatric Patients (N = 64) on Extracorporeal Membrane Oxygenation from 2018 to 2022: A Single-Center Report
Source: Nutrients. 2023 Jul 20;15(14):3221. doi: 10.3390/nu15143221 (PMC10383656; doi:10.3390/nu15143221)
Supplement: Supplementary file 1 [file nutrients-15-03221-s001.zip › Supplementary Information S1.pdf]

Supplemental

Supplemental information S1:

The RN must choose one of the following when admitting the patient, it is part of the Pediatric Patient Profile that they fill out:

Nutrition Risk Screen
No indicators present
NPO/clear liquid greater than 5 days
Difficulty chewing/swallowing
Failure to grow or gain weight
Feeding intolerance (excluding gastroenteritis)
Large or nonhealing wound, burn or pressure injury
<50% oral intake greater than 5 days
Tube feeding or parenteral nutrition
Supplemental drinks consumed at home
Unintentional weight loss
Other (see comments)

Medical Nutrition Therapy Standards of Care booklet.

Reasons for Completing Nutrition Assessments:

- Enteral Feeds or TPN/PPN
- NPO/CL ≥ 5 consecutive days
- Calorie Count
- Difficulty Chewing/Swallowing (that is impacting nutritional intake)
- Unintentional Weight Loss
- ≤ 50% usual intake for 5 days
- Admitting Diagnosis
    - Inborn Errors of Metabolism
    - Cystic Fibrosis
    - Failure to Thrive
    - Burns
    - Bone Marrow Transplant
    - Ketogenic Diet
    - Eating Disorder

For normal advancement
If weight is less than 3 kg, begin at 1 mL per hour and advance by 1 mL per hour every 6 hours.
If weight is greater than 3 kg, begin at 2 mL per hour and advance by 2 mL per hour every 6 hours.

For high-risk advancement
If weight is less than 3 kg, begin at 1 mL per hour and advance by 1 mL per hour every 12 hours
If weight is greater than 3 kg, begin at 1 mL per hour and advance by 1 mL per hour every 6 hours.

DRI reference: Dietary Intakes, Institute of Medicine of the National Academies

Infants and Young Children

Estimated Energy Requirements (kcal/day) = Total Energy Expenditure + Energy Deposition

0-3 months	EER = (89 x weight [kg] -100) + 175
4-6 months	EER = (89 x weight [kg] -100) + 56
7-12 months	EER = (89 x weight [kg] -100) + 22
13-35 months	EER = (89 x weight [kg] -100) + 20

Supplemental Table S1. Nutrition Information

Variable	Overall (N=64)
Feed Type Same Across ECMO Days	18 (28.1)
Calories Present	48 (75.0)
Calories Total	1942.69 [456.90, 3844.40]
Protein Present	48 (75.0)
Protein Total	73.78 [22.55, 188.94]
Proportion of Days on Extra Protein	50.00 [21.11, 73.86]
Proportion of Days Calorie Goal Met	0.00 [0.00, 0.00]
Proportion of Days Calories Underfed	100.00 [97.73, 100.00]
Proportion of Days Protein Goal Met	N=63; 33.33 [0.00, 56.25]
Proportion of Days Protein Underfed	63.07 [43.30, 100.00]

Note: The proportion of days calories or protein underfed are 100 because everyone had a 100% that were underfed across the days. Numeric data is expressed as median [25th, 75th percentile] and categorical data is expressed as count (percent).

Supplemental Table S2. Nutrition Information by Mortality

- The proportion of days calories or protein underfed are 100 because everyone had a 100% that were underfed across the days.

Variable	Alive 28-day (N=36)	Deceased 28-day (N=28)	*p*-value
Feed Type Same Across ECMO Days	8 (22.2)	10 (35.7)	0.2337
Parenteral	2 (25.0)	2 (20.0)	
No Nutrition Support	6 (75.0)	8 (80.0)	
Calories/Proteins Present	29 (80.6)	19 (67.9)	0.2445
Calories Total	2129.30 [400.50, 4003.20]	1854.30 [513.30, 3423.04]	0.6129
Protein Total	63.39 [20.70, 195.68]	84.25 [24.40, 173.70]	0.8661
Proportion of Days on Extra Protein	43.56 [25.00, 66.67]	50.00 [17.71, 100.00]	0.4498
Proportion of Days Calorie Goal Met	0.00 [0.00, 2.27]	0.00 [0.00, 0.00]	0.3306
Proportion of Days Protein Goal Met	N=35<br>27.27 [0.00, 54.55]	N=28<br>33.33 [0.00, 57.67]	0.9659
Overall Percent of Calorie Goal (including patients with 0)	35.1 [5.7, 49.7]	28 [1.5, 51.2]	0.9396
Percent of Calorie goal (patients that received > 0 of goal)	41.63 [19.15, 50.42]	41.1 [23.2, 55.2]	1.0

Notes: Numeric data is expressed as median [25th, 75th percentile] and categorical data is expressed as count (percent).

Models

Supplemental Table S3. Multiple Linear Regression Model – Percent Feed Goal Met

Variables below not included in final model due to insignificance

Summary of Backward Elimination		
**Step**	**Label**	***p*- Value**
1	Were there any ECMO complications?	0.9065
2	Feeding intolerance	0.7853
3	Congenital Heart Disease	0.6782
4	Prematurity	0.6478
5	G or J-tube dependence	0.6592
6	Failure to thrive	0.4063
7	Chromosomal abnormality	0.3717
8	Cardiac arrest in the 24 hours prior to ECMO initiation?	0.3400
9	Prior bowel surgery	0.3562
10	Neonatal vs. Pediatric	0.1316

**Model Summary**	
Number of Observations Read	64
Number of Observations Used	60
Number of Observations with Missing Values	4
Adjusted R-Square	0.1004

Supplemental Table S4. Regression models with Outcomes – Percent Feed Goal Met (Independent Variable)

**Logistic Regression**	**n**	**Odds Ratio**	***p*-value**
28 day Mortality (Yes)	60	0.997 (0.975, 1.020)	0.8139

Summary:

- Model 1: Logistic regression with 28 day Mortality as the outcome and Percent Calorie Goal met as the predictor was modeled. The model did not find any significant association between the percent of the calorie goal met and 28 day mortality.

**Simple Linear Regression**	N	**Estimate**	**% change**	***p*-value**
Hospital Length of Stay	59	0.01 (-0.01, 0.02)	0.6 (-0.6, 1.8)	0.3149
PICU Length of Stay	60	0.01 (0, 0.02)	0.8 (-0.5, 2.2)	0.2128
Days Mechanical Ventilation	34	0.01 (-0.01, 0.04)	1.5 (-0.8, 3.8)	0.2064

Summary:

- Model 2: Linear regression with Hospital Length of Stay as the outcome and Percent calorie goal met as the predictor did not find any statistically significant association between hospital length of stay and the percent of the calorie goal met.
- Model 3: Linear regression with PICU Length of Stay as the outcome and Percent calorie goal met as the predictor did not find any statistically significant association between PICU length of stay and the percent of the calorie goal met.
- Model 4: Linear regression with Days Mechanical Ventilation as the outcome and Percent calorie goal met as the predictor did not find any statistically significant association Days Mechanical Ventilation and the percent of the calorie goal met.
