# Supplementary material for: Daily Nutritional Intake of Pediatric Patients (N = 64) on Extracorporeal Membrane Oxygenation from 2018 to 2022: A Single-Center Report
Source: Nutrients. 2023 Jul 20;15(14):3221. doi: 10.3390/nu15143221 (PMC10383656; doi:10.3390/nu15143221)

Enteral Feeding Guideline

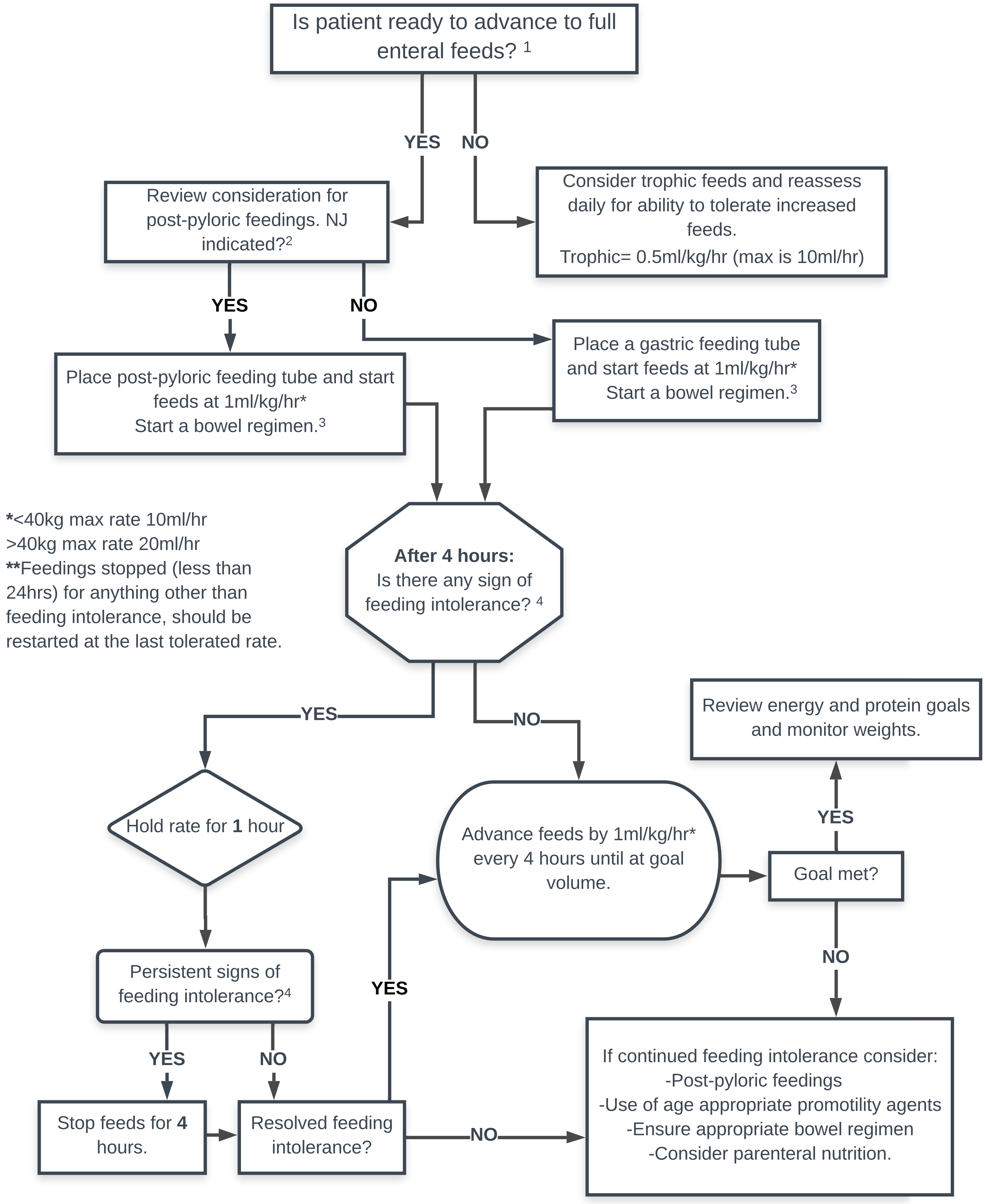

Supplement: Supplementary file 1 [file nutrients-15-03221-s001.zip › Supplementary Information S2.pdf]
