# Supplementary material for: Daily Nutritional Intake of Pediatric Patients (N = 64) on Extracorporeal Membrane Oxygenation from 2018 to 2022: A Single-Center Report
Source: Nutrients. 2023 Jul 20;15(14):3221. doi: 10.3390/nu15143221 (PMC10383656; doi:10.3390/nu15143221)
Supplement: Supplementary file 1 [file nutrients-15-03221-s001.zip › Supplementary Information S3.pdf]

Enteral Feeding Guideline

[1]Contraindications to Enteral Nutrition

Definite contraindications to enteral nutrition

-Escalating vasoactive or inotropic support
-Hemodynamic instability with ongoing volume rescucitation
-Suspected or confirmed necrotizing enterocolitis or intestinal ischemia
-Mechanical bowel obstruction
-Significant gastrointestinal bleeding

Relative contraindications to enteral nutrition

-First 24 hours after cardiac surgery or open chest
-Ongoing central hypothermia
-Less than 24 hours after cardiac arrest
-Severe ileus with high nasogastric output and/or emesis
-High gastrointestinal stomal output
-Intractable diarrhea

[2]When to consider post-pyloric feeds?

Aspiration Risk

-Previous history of aspiration
-Witnessed regurgitation or aspiration of gastric contents
-Severe gastro-esophageal reflux disease
-Altered mental status with depressed gag and cough reflexes.
-Persistent vomiting (2 or more episodes in a 24-hour period).
-Noninvasive ventilation **WITH** escalating or high settings.

NPO for greater than 24 hours and...

-anticipate the patient will remain on respiratory support and not safe for NG feedings. Do not start feedings if respiratory status remains unstable and patient may need intubation.

[3]Bowel Regimen

Osmotic laxative: Miralax, Lactulose
Stimulant laxative: Senna, Bisacodyl
Emollient laxative (softener): Docusate

Rectal options: glycerin suppository, docusate suppository, sodium phosphate enema

[4]Signs and Symptoms of Intolerance

-Vomiting (2 or more episodes/24 hours)
-Abdominal discomfort with distention and tenderness
-Diarrhea- 3 or more episodes of loose stool in 24 hours not related to antibiotics or a bowel regimen.
**There is no evidence to support the use of abdominal girth or gastric residual as a sign of feeding intolerance.*

Nutritional Goals

Age	Kcal/Kg/day
0-3 mo	102
4-36 mo	82
4-6 yrs	65
7-10 yrs	55
11-18 yrs	40

* if the patient is mechanically ventilated, subtract 10% of the total daily calories. Do not subtract for NIPPV.

NPO time for Procedures

-All tube feedings must be stopped 8 hours prior to scheduled surgery/sedation (Breastmilk can be held 6 hours prior).
-Intubated patients with post-pyloric feeds do not need to have feedings stopped. (If extubating, stop post-pyloric feedings 8hrs prior).
-Clear liquids and medications may be given in all types of tubes until 2 hours prior to scheduled surgery (broth and breastmilk are not considered clear).
