# Supplementary material for: Daily Nutritional Intake of Pediatric Patients (N = 64) on Extracorporeal Membrane Oxygenation from 2018 to 2022: A Single-Center Report
Source: Nutrients. 2023 Jul 20;15(14):3221. doi: 10.3390/nu15143221 (PMC10383656; doi:10.3390/nu15143221)
Supplement: Supplementary file 1 [file nutrients-15-03221-s001.zip › Supplementary Information S4.pdf]

Initiate Enteral Feeds

After assessing feeding readiness, use this information to guide initiation of enteral feeds. Determine type, route and rate and volume and advance while monitoring tolerance to goal of 150 mL/kg/day.

Readiness Assessment includes:

Adequate cardiac output
Stable hemodynamics, weaning inotropic support
Stable rhythm or stable arrhythmia
No clinical seizure activity for > 12 hours
No signs/symptoms of NEC or gut ischemia

**Step 1**<br>**Determine Type of Feed**	Human milk or donor milk preferred, based on mother's feeding plan<br>If human milk is not available or parent preference is to use formula:<br>≥ 37 weeks – Enfamil® Infant 20 cal/oz, Good Start® Gentle 20 cal/oz, Similac® Advance® 19 cal/oz<br>≥ 35 to < 37 weeks – Enfamil® Enfacare® 22 cal/oz, Similac® Expert Care NeoSure® 22 cal/oz	
**Step 2**<br>**Determine Route**	BOLUS<br><br>PO + NG	Patients demonstrating **oral feeding readiness** based on these guidelines:<br>Patient on ≤ 2 L NC<br>Resp rate < 70 breaths per minute<br>Demonstrating feeding cues:<br>○ Awake/alert<br>○ Rooting<br>○ Sucking on pacifier<br>○ Hands to mouth<br>Document feeding cues in EPIC Intake Flowsheet.
	CONTINUOUS<br><br>NG or ND[1]	Patient does not demonstrate oral feeding readiness as listed above.<br>Conditions that require post-pyloric feeds:<br>Congenital GI anomalies<br>Known gastroparesis<br>Severe GERD<br>High-aspiration risk<br>Ileus

1. Patients without conditions that require post-pyloric feeds in whom ND cannot be placed at the bedside, may attempt continuous NG feeds and consider IR placement of post-pyloric ND tube.

Step 3 Determine Appropriate Rate and Advance			
	**Continuous (NG or ND)**	**Bolus (PO + NG)**	**Goal***
**Normal Advance**<br>• **Patients ≥ 3 days of age**	**< 3 kg**<br>○ Initiate at 1 mL/hr<br>○ Advance by 1 mL/hr q 6 hr to goal*<br>**≥ 3 kg**<br>○ Initiate at 2 mL/hr<br>○ Advance by 2 mL/hr q 6 hr to goal*	Initiate at 15 mL q 3 hr<br>Advance by 5 mL q 6 hr to goal<br>Please add in the diet order comments the dot phrase **.cardiacpoadlib**[2]	Advance to goal of<br>150 mL/kg/day
**Slow Advance**<br>• **< 37 weeks gestation**<br>• **Patients < 3 days of age**<br>• **Ready to advance from NPO or trophic feeds**	**< 3 kg**<br>○ Initiate at 1 mL/hr<br>○ Advance by 1 mL/hr q 12 hr to goal*<br>**≥ 3 kg**<br>○ Initiate at 1 mL/hr<br>○ Advance by 1 mL/hr q 6 hr to goal*	Initiate at 5 mL q 3 hr<br>Advance by 5 mL q 6 hr to goal<br>Please add in the diet order comments the dot phrase **.cardiacpoadlib**[2]	

2. **.cardiacpoadlib:** Minimum feed volume is *** mL every 3 hrs. Infant may take more than minimum volume. Feeding frequency based on infant's cues. Do not go longer than 3 hours without offering a feed. Notify ordering provider if not taking minimum feed volume.

Advancing (or Titrating) PN While Advancing Enteral Nutrition:

- **When enteral feeds are initiated, any enteral volume administered may exceed the intravenous TFL of 100 mL/kg/day up to 130 mL/kg/day prior to weaning PN.**
- Continue IV lipids until enteral caloric intake ≥ 100 kcal/kg/day.
- Once goal enteral nutrition achieved, discontinue TFL order.
- Discuss/evaluate TFL order daily.

Advancing to Goal Calories

- Goal volume of 150 mL/kg/day will provide minimum requirement of 100 kcal/kg/day.
- Once goal volume is reached, assess growth and determine further caloric goals with dietician. Infants may require a further increase in volume, hindmilk and/or fortification (See Fortification Guidelines).

Ongoing Monitoring and Fortification Guidelines

Ongoing Monitoring

**Daily**	Weight<br>Actual calorie intake (review on rounds)<br>Goal calories ordered (review on rounds and compare with actual calorie intake)
**Weekly**	Head circumference<br>Length
**NG Tube**	Leave in place until neonate meets goal calories by mouth for 2 consecutive days.<br>If exclusively breastfeeding, may remove NG tube if the mother's milk supply is established per lactation consultant and the infant is breastfeeding a minimum of 8 times per day for 2 consecutive days.
**Poor Weight Gain**	Consult RD prior to fortification<br>See fortification guidelines

Fortification Guidelines

**Indications for Fortification**	If infant is unable to reach the calorie goal due to volume restrictions.<br>If infant has lost more than 10% of birth weight by 14 days of life.<br>If infant cannot tolerate ≥ 160 mL/kg/day of breast milk.<br><br>Please Note:<br><br>Do not recommend fortification at less than 14 days of age unless recommended by RD.<br>Weigh the risk/benefit of fortifying before 14 days of age.
**Instructions for Fortification**	Consider hindmilk for fortification (discuss with lactation consultant).<br>○ Separating Your Milk to Help Your Baby Grow (32:B:21)<br>Fortify to 22 kcal/oz after 24 hours of tolerating goal volume.<br>Advance to 24 kcal/oz, if needed, after 24 hours of tolerating 22 kcal/oz.<br>Do not increase to 27 kcal/oz unless discussed with RD.<br>Inpatient fortification: Fortification with formula liquid concentrate preferred for safety and sterility in formula room/Human Milk Management Center.<br>Use standard infant formula for fortification, unless otherwise indicated by RD.

Post-op's that require slow advancement/high risk protocol:

s/p Norwood, any pt with moderate to severely depressed cardiac function

Pre-op's that require TPN and only ad lib or trophic feeds (no advancement):

Ductal dependent (pulmonary): Single ventricle physiology (HLHS), truncus arteriosis, APW, critical aortic stenosis, interrupted aortic arch.

Ductal dependent (systemic): TOF, tricuspid atresia, pulmonary atresia, pulmonary stenosis, severe Ebstein's anomaly
